# Development and external validation of a dynamic nomogram for predicting the risk of functional outcome after 90 days in patients with acute intracerebral hemorrhage

**DOI:** 10.3389/fneur.2025.1519091

**Published:** 2025-01-29

**Authors:** Shaojie Li, Hongjian Li, Jiani Chen, Baofang Wu, Jiayin Wang, Chaocan Hong, Changhu Yan, Weizhi Qiu, Yasong Li, Hongzhi Gao

**Affiliations:** ^1^Department of Neurosurgery, The Second Affiliated Hospital of Fujian Medical University, Quanzhou, Fujian, China; ^2^Department of Radiology, Affiliated Hospital of North Sichuan Medical College, North Sichuan Medical College, Nanchong, China; ^3^Department of Neurosurgery, Jinjiang Hospital of Traditional Chinese Medicine, Quanzhou, China

**Keywords:** intracerebral hemorrhage, dynamic nomogram, external validation, prediction, prognosis

## Abstract

**Background and purpose:**

Intracerebral hemorrhage remains a significant cause of death and disability worldwide, highlighting the urgent need for accurate prognostic assessments to optimize patient management. This study aimed to develop a practical nomogram for risk prediction of poor prognosis after 90 days in patients with intracerebral hemorrhage.

**Methods:**

A retrospective study was conducted on 638 patients with intracerebral hemorrhage in the Second Hospital of Fujian Medical University, China, who were divided into a training set (*n* = 446) and a test set (*n* = 192) by random splitting. Then the data on demographics, clinical symptoms, imaging characteristics, and laboratory findings were collected. In this study, adverse outcomes were defined as a Modified Rankin Scale (mRS) score of 3–6 at 90 days post-ICH onset, as assessed during follow-up. Later, least absolute shrinkage and selection operator (LASSO) regression and multifactorial logistic regression were used to screen the variables and construct a nomogram. Next, the evaluation was performed using the Receiver Operating Characteristic (ROC) curve, calibration curve, and decision curve analysis. Finally, the external validation was completed using the data of 496 patients with intracerebral hemorrhage from the Jinjiang Hospital of Traditional Chinese Medicine.

**Results:**

In the training and test sets of intracerebral hemorrhage, the incidence of poor prognosis was 60.53 and 61.46%, respectively. Through variable screening, this study identified age, Glasgow Coma Scale (GCS), blood glucose, uric acid, hemoglobin, and hematoma location as independent predictors of poor prognosis in intracerebral hemorrhage. The developed dynamic nomogram was easy to use and demonstrated strong predictive performance (training set AUC: 0.87; test set AUC: 0.839; external validation set AUC: 0.774), excellent calibration, and clinical applicability.

**Conclusion:**

The dynamic nomogram we developed using five independent risk factors serves as a practical tool for real-time risk assessment and can help facilitate early intervention and personalized patient management, thereby improving clinical outcomes in high-risk patients.

## Introduction

Primary Intracerebral Hemorrhage (ICH) is a subtype of stroke involving non-traumatic bleeding within the brain parenchyma and accounts for nearly one-third of all stroke incidences in China, following ischemic stroke (1, 2). ICH is not only a leading cause of death and long-term disability worldwide but also imposes substantial economic and social burdens (3). A nationwide stroke survey reveals that the burden of stroke in China has continuously risen over recent decades. The prognosis for ICH is particularly poor, with a mortality rate of about 40% within 1 month after onset, which may increase to 54% within 1 year, and only a 24% survival rate at 3–5 years. For those who survive the initial event, only 12–39% regain functional independence for basic daily activities (4). Despite advancements in surgical interventions such as craniotomy for hematoma evacuation, stereotactic surgery, particularly endoscopic techniques, and specialized stroke unit care showing treatment efficacy, the rate of poor outcomes remains high (5–7). Over recent decades, researchers have increasingly focused on identifying prognostic biomarkers to accurately identify individuals at high risk of poor outcomes (8–10). By prioritizing these individuals for admission to intensive care units and implementing early personalized management, the prognosis of ICH patients can be significantly improved.

Previous studies have shown that the prognosis of ICH is influenced by a variety of factors, including demographic characteristics, hematoma volume, hemorrhage site, and inflammatory response (11). Despite the proliferation of predictive models for the prognosis of intracerebral hemorrhage, these models often exhibit significant limitations. For instance, while models utilizing radiological features from NCCT imaging of hematoma and perihematomal tissue demonstrate good predictive accuracy, their complexity restricts practical clinical application (12). Furthermore, many models based on readily available clinical indicators frequently lack external validation, which limits their generalizability and broad applicability (13–15). Additionally, some studies have developed predictive models but have not integrated them into practical platforms, thereby impacting their clinical utility (16, 17).

In light of this, our study aimed to integrate various clinical characteristics to accurately predict ICH patients’ short-term prognosis and successfully developed a streamlined and efficient predictive platform. This platform not only enhances the usability of the predictive model but also, through external validation, has demonstrated its effectiveness and reliability across diverse clinical settings, significantly enhancing its clinical utility.

## Materials and methods

### Study population

**Figure 1:**
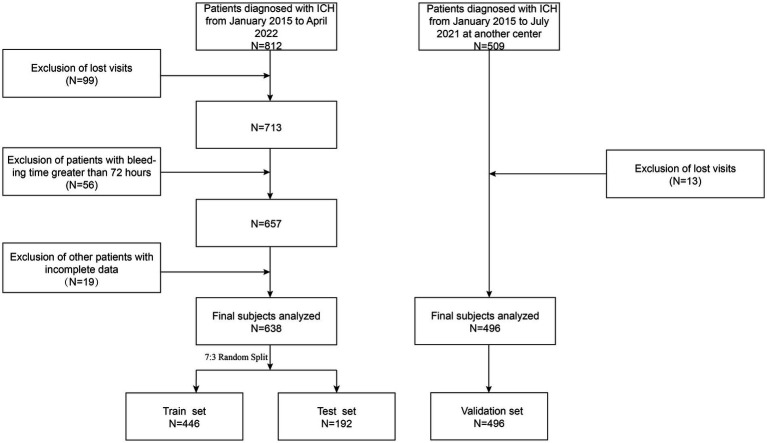
Participant inclusion flowchart.

This study is a retrospective analysis of one center of patients with intracerebral hemorrhage treated in the Department of Neurosurgery at the Second Affiliated Hospital of Fujian Medical University from January 2015 to April 2022. The study defined training and validation cohorts using specific inclusion and exclusion criteria. The inclusion criteria included: (1) age over 18 years; (2) hemorrhagic stroke confirmed by computed tomography (CT); (3) admission within 72 h after symptom onset; (4) first acute ICH. The exclusion criteria included: (1) traumatic intracerebral hemorrhage; (2) Secondary cerebral hemorrhage (e.g., post-infarction cerebral hemorrhage, vascular malformation, aneurysm, tumor stroke); (3) significant systemic diseases (such as cardiac, liver, renal insufficiency, or hematological diseases); (4) incomplete clinical or imaging data. To validate these findings, an external validation cohort at the Jinjiang Hospital of Traditional Chinese Medicine was established using the same exclusion criteria, covering patients with intracerebral hemorrhage from January 2015 to July 2021. Figure 1 shows the results of the participant screening. The study was approved by the Second Affiliated Hospital of Fujian Medical University (2022–35) and the Jinjiang Hospital of Traditional Chinese Medicine (2022–32), and informed consent was waived in this study.

### Data collection

Utilizing the hospital’s electronic information system, we gathered essential data on patients admitted with intracerebral hemorrhage, including demographic variables such as age and gender, medical history, laboratory results, radiographic data, and treatment details. Baseline characteristics related to medical history included body temperature, systolic and diastolic blood pressure, conditions such as hypertension, diabetes, smoking, drinking history, and prior anticoagulant, or antiplatelet therapy. The clinical neurological status of patients was assessed using the Glasgow Coma Scale (GCS). As for intubated and/or sedated patients, pre-intubation and post-resuscitation GCS scores were documented. Recorded imaging features of ICH included the presence of intraventricular hemorrhage (IVH), specific location and volume of the hematoma, and extent of midline shift. Hematoma locations were categorized into superficial (originating at the cortex-subcortex junction) and deep (e.g., basal ganglia, thalamus, cerebellum, or brainstem) (18). Hematoma volume was calculated using the Tada formula (19). Laboratory tests included counts of white blood cells, neutrophils, monocytes, lymphocytes, platelets, hemoglobin, albumin, uric acid, glucose, activated partial thromboplastin time (APTT), international normalized ratio (INR), prothrombin time (PT), fibrinogen, and thrombin time. Inflammatory markers were used to compute ratios such as platelet-to-lymphocyte ratio (PLR), neutrophil-to-lymphocyte ratio (NLR), and lymphocyte-to-monocyte ratio (LMR) (20). Early hematoma growth (uHG) was defined as the initial ICH volume (ml) divided by the time from onset to baseline CT scan (hours) (21). Interventional treatments included surgery and tracheotomy. Previous studies suggest these indicators may correlate with survival outcomes in patients with intracerebral hemorrhage.

### Outcome evaluation

Functional outcomes were assessed in all patients by outpatient and telephone follow-up for modified Rankin Scale (mRS) scores 90 days after the ICH episode. Each patient provided at least two contact numbers. For those unreachable, attempts were made to contact them once a week over 3 weeks. The follow-up team received specialized training to ensure the consistency of the data. The primary clinical outcome was assessed 90 days post-ICH onset, focusing on adverse outcomes defined as death or severe disability, represented by a Modified Rankin Scale (mRS) score of 3–6 (22, 23).

### Statistical analysis

Categorical variables were expressed as percentages, while continuous variables were described using means and standard deviations (SD). Differences between groups were determined using the *t*-test, Mann–Whitney *U* test, chi-square test, and Fisher’s exact test (24). The dataset was randomly divided into a training set and a test set in a ratio of 7:3. In the training set, feature selection for 33 variables was conducted using the least absolute shrinkage and selection operator (LASSO), and 22 features corresponding to one standard error of the optimal *λ* value were included in a multivariable regression analysis to identify independent prognostic factors (25). The Variance Inflation Factor (VIF) was used to assess multicollinearity. For clinical applicability, the identified independent risk factors were used to construct an accessible dynamic web-based nomogram. Then the reliability of the nomogram was evaluated using the Receiver Operating Characteristic (ROC) curve and its Area Under the Curve (AUC). Later, the model calibration was performed using the Hosmer-Lemeshow goodness-of-fit test and calibration plots from 1,000 bootstrap resamples. Furthermore, the model’s clinical utility was assessed using Decision Curve Analysis (DCA). ROC AUC, Hosmer-Lemeshow test, calibration curves, and clinical decision curves were applied in an internal test set and validated in an external validation cohort to comprehensively evaluate the nomogram’s performance. All statistical analyses were performed using R version 4.3 and Python version 3.12.0.

## Results

### Baseline characteristics

In this study, 638 patients were included and the dataset was randomly divided into a training set and a test set. The training set comprised 446 patients for model training, while the test set included 192 patients for model validation. The average age of the patients was 58.50 years, with males constituting 64.58% (412 patients) and females 35.42% (226 patients). The incidence of adverse outcomes within 90 days was 60.81% overall, with 60.53% in the training set and 61.46% in the test set. Table 1 shows the detailed baseline characteristics of patients’ GCS scores, vital signs, and laboratory indicators. Furthermore, this study divided the baseline characteristics of the poor prognosis population by sex (Supplementary Table S1). Among patients with poor prognosis for acute intracerebral hemorrhage, men typically had higher diastolic blood pressure, uric acid levels, hemoglobin concentration, APTT values, and higher risk of smoking and alcohol consumption than women. In addition, men have lower levels of inflammation, as evidenced by lower LMR and PLR.

**Table 1:** Table of baseline information on participants’ training and test sets.

Variable names	Train			Test			
	Favorable outcome group	Unfavorable outcome group	*P*	Favorable outcome group	Unfavorable outcome group	*P*	Total *P*
	*N* = 176	*N* = 270		*N* = 74	*N* = 118		
Age (years)	58.43 ± 10.39	58.29 ± 10.89	0.892	58.84 ± 10.70	58.86 ± 10.07	0.986	0.574
GCS	12.48 ± 2.27	8.75 ± 2.62	<0.001	12.55 ± 2.10	9.27 ± 2.62	<0.001	0.233
Temperature (°C)	36.701 ± 0.47	36.99 ± 0.71	<0.001	36.82 ± 0.54	36.86 ± 0.62	0.664	0.584
Systolic blood pressure (mmHg)	152.71 ± 17.33	166.20 ± 20.66	<0.001	155.37 ± 18.64	164.01 ± 20.39	0.004	0.909
Diastolic blood pressure (mmHg)	88.02 ± 12.83	92.22 ± 15.28	0.003	87.12 ± 13.03	91.26 ± 15.58	0.058	0.479
Glucose (mmol/L)	7.39 ± 2.33	8.62 ± 3.19	<0.001	7.46 ± 2.23	8.83 ± 3.93	0.007	0.528
Uric acid (μmol/L)	289.89 ± 100.37	309.46 ± 133.28	0.097	286.45 ± 114.42	321.98 ± 140.63	0.069	0.544
Albumin (g/L)	41.22 ± 3.95	41.27 ± 4.26	0.898	40.37 ± 4.24	40.72 ± 4.32	0.582	0.064
Leucocyte	8.67 ± 2.89	10.33 ± 4.18	<0.001	9.01 ± 3.52	10.72 ± 4.49	0.006	0.251
Hemoglobin (g/L)	154.06 ± 20.16	152.14 ± 24.80	0.393	147.99 ± 19.23	152.99 ± 22.04	0.110	0.345
NLR	8.54 ± 6.05	10.46 ± 8.64	0.011	8.32 ± 7.21	11.41 ± 8.67	0.011	0.448
LMR	3.41 ± 2.12	3.34 ± 3.06	0.793	3.59 ± 2.82	3.10 ± 3.30	0.295	0.730
PLR	195.57 ± 97.64	189.69 ± 117.80	0.582	178.60 ± 89.27	189.76 ± 117.60	0.485	0.488
PT	11.53 ± 1.82	11.61 ± 2.31	0.673	11.59 ± 1.12	12.31 ± 4.54	0.183	0.050
INR	1.02 ± 0.17	1.03 ± 0.22	0.575	1.02 ± 0.10	1.09 ± 0.40	0.150	0.071
APTT	24.96 ± 5.37	25.36 ± 5.25	0.435	25.59 ± 4.78	25.89 ± 6.44	0.730	0.225
D-dimer	2.01 ± 10.31	1.58 ± 3.62	0.534	2.04 ± 8.26	2.17 ± 11.87	0.931	0.051
TT	17.23 ± 2.46	16.91 ± 2.54	0.186	16.67 ± 2.69	16.71 ± 2.88	0.928	0.132
FIB	2.867 ± 0.70	2.91 ± 0.96	0.578	3.09 ± 1.34	3.03 ± 0.84	0.694	0.604
Bleeding volume (mL)	12.17 ± 11.71	34.65 ± 26.37	<0.001	11.27 ± 8.5	30.36 ± 23.91	<0.001	0.172
uHG	3.61 ± 3.73	10.94 ± 9.11	<0.001	3.17 ± 3.03	9.94 ± 8.42	<0.001	0.304
Sex (%)	111 (63.07)	182 (67.41)	0.400	38 (51.35)	81 (68.64)	0.024	0.418
Male	111 (63.07)	182 (67.41)		38 (51.35)	81 (68.64)		
Female	65 (36.93)	88 (32.59)		36 (48.65)	37 (31.36)		
Hypertension (%)	64 (36.36)	92 (34.07)	0.694	28 (37.84)	26 (22.03)	0.027	0.110
No	64 (36.36)	92 (34.07)		28 (37.84)	26 (22.03)		
Yes	112 (63.64)	178 (65.93)		46 (62.16)	92 (77.97)		
Diabetes (%)	156 (88.64)	220 (81.48)	0.058	68 (91.89)	92 (77.97)	0.020	0.850
No	156 (88.64)	220 (81.48)		68 (91.89)	92 (77.97)		
Yes	20 (11.36)	50 (18.52)		6 (8.11)	26 (22.03)		
Smoking (%)	160 (90.91)	235 (87.04)	0.270	69 (93.24)	104 (88.14)	0.365	0.665
No	160 (90.91)	235 (87.04)		69 (93.24)	104 (88.14)		
Yes	16 (9.09)	35 (12.96)		5 (6.76)	14 (11.86)		
Drinking (%)	166 (94.32)	245 (90.74)	0.233	66 (89.19)	104 (88.14)	1.000	0.188
No	166 (94.32)	245 (90.74)		66 (89.19)	104 (88.14)		
Yes	10 (5.68)	25 (9.26)		8 (10.81)	14 (11.86)		
History of anticoagulant use (%)	168 (95.45)	250 (92.59)	0.309	72 (97.30)	114 (96.61)	1.000	0.152
No	168 (95.45)	250 (92.59)		72 (97.30)	114 (96.61)		
Yes	8 (4.55)	20 (7.41)		2 (2.70)	4 (3.39)		
Tracheotomy (%)	165 (93.75)	195 (72.22)	<0.001	69 (93.24)	93 (78.81)	0.013	0.324
No	165 (93.75)	195 (72.22)		69 (93.24)	93 (78.81)		
Yes	11 (6.25)	75 (27.78)		5 (6.76)	25 (21.19)		
Lateral ventricular hemorrhage (%)	141 (80.11)	189 (70.00)	0.023	66 (89.19)	81 (68.64)	0.002	0.558
No	141 (80.11)	189 (70.00)		66 (89.19)	81 (68.64)		
Yes	35 (19.89)	81 (30.00)		8 (10.81)	37 (31.36)		
Location of hematoma (%)	57 (32.39)	36 (13.33)	<0.001	25 (33.78)	18 (15.25)	0.005	0.740
Deep-seated hematoma	57 (32.39)	36 (13.33)		25 (33.78)	18 (15.25)		
Superficial hematoma	119 (67.61)	234 (86.67)		49 (66.22)	100 (84.75)		
Centerline shift (%)	172 (97.73)	182 (67.41)	<0.001	72 (97.30)	82 (69.49)	<0.001	0.894
No	172 (97.73)	182 (67.41)		72 (97.30)	82 (69.49)		
Yes	4 (2.27)	88 (32.59)		2 (2.70)	36 (30.51)		
Surgeries (%)			<0.001			<0.001	0.062
No	133 (75.57)	85 (31.48)		59 (79.73)	51 (43.22)		
Yes	43 (24.43)	185 (68.52)		15 (20.27)	67 (56.78)		

GCS, Glasgow Coma Scale; NLR, neutrophil-to-lymphocyte ratio; LMR, lymphocyte-to-monocyte ratio; PLR, platelet-to-lymphocyte ratio; PT, prothrombin time; INR, international normalized ratio; APTT, activated partial thromboplastin time; TT, thrombin time; FIB, fibrinogen; uHG, ultraearly hematoma growth.

### LASSO regression analysis combined with multifactor logistic regression analysis to screen predictor variables

**Figure 2:**
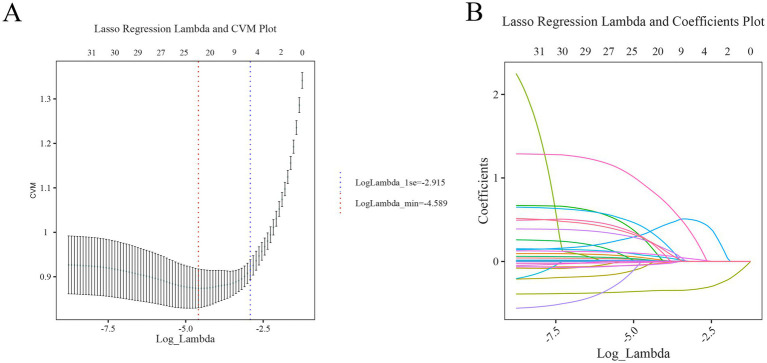
Initial screening process of LASSO regression analysis **(A)** cross-validation curve: the red and blue vertical dashed lines indicate the Log(*λ*) corresponding to one standard error difference from the minimum error (lambda.min), respectively. **(B)** LASSO path diagram: the regression coefficients versus Log(λ) as the coefficient scores gradually decrease.

This study identified outcome-related features through combining LASSO regression and multivariable logistic regression analysis. LASSO regression, which reduces model overfitting by shrinking coefficients, was utilized to select features, as depicted in Figures 2A,B. Based on the 23 non-zero coefficients corresponding to lambda.min (Supplementary Table S2), we identified predictors including age, hypertension, smoking, drinking, Glasgow Coma Scale (GCS), body temperature, systolic blood pressure, glucose, uric acid, albumin, white blood cells, hemoglobin, lymphocyte-to-monocyte ratio (LMR), platelet-to-lymphocyte ratio (PLR), activated partial thromboplastin time (APTT), thrombin time (TT), D-dimer, tracheotomy, hematoma location, hematoma volume, midline shift, ultra early hematoma growth (uHG), and surgical treatment. To minimize the impact of confounding variables, these 23 predictors were included in the multivariable logistic regression analysis. Ultimately, five key variables, such as GCS, glucose, uric acid, hemoglobin, and hematoma location, were statistically significant in poor prognosis (Table 2), and there was no considerable covariance among the variables (VIF < 2) (Supplementary Table S3). Because of this, GCS, glucose, uric acid, hemoglobin, and hematoma locations were included in this study for modeling.

**Table 2:** Multifactorial logistic regression analysis of poor prognosis in intracerebral hemorrhage.

Variable name	OR	95% CI	*P*-value
(Intercept)	0.00	−32.94,9.55	0.29
Hypertension	0.76	−0.86,0.31	0.36
Smoking	1.72	−0.30,1.42	0.21
Drinking	2.05	−0.33,1.81	0.19
GCS	0.68	−0.53,-0.25	**0.00**
Temperature	1.51	−0.13,0.97	0.14
Systolic blood pressure	1.02	0.00,0.03	0.07
Glucose	1.12	0.02,0.22	**0.02**
Uric acid	1.00	0.00,0.00	**0.04**
Albumin	0.94	−0.14,0.01	0.09
Leucocyte	1.10	0.00,0.19	0.06
Hemoglobin	0.98	−0.04,-0.01	**0.01**
LMR	1.06	−0.03,0.18	0.21
PLR	1.00	0.00,0.00	0.17
APTT	1.04	−0.02,0.11	0.19
TT	0.93	−0.20,0.06	0.30
Tracheotomy	1.98	−0.18,1.61	0.13
Deep-seated hematoma	3.54	0.55,2.02	**0.00**
Centerline shift	1.64	−0.70,1.88	0.44
Bleeding volume	1.00	−0.04,0.05	0.97
uHG	1.13	0.00,0.26	0.06
D-dimer	1.01	−0.03,0.04	0.49
Surgeries	1.15	−0.56,0.82	0.70

GCS, Glasgow Coma Scale; LMR, lymphocyte-to-monocyte ratio; PLR, platelet-to-lymphocyte ratio; APTT, activated partial thromboplastin time; TT, thrombin time; uHG, ultraearly hematoma growth. *P* values bolded indicate that the variable is statistically significant.

### Construction of predictive models

**Figure 3:**
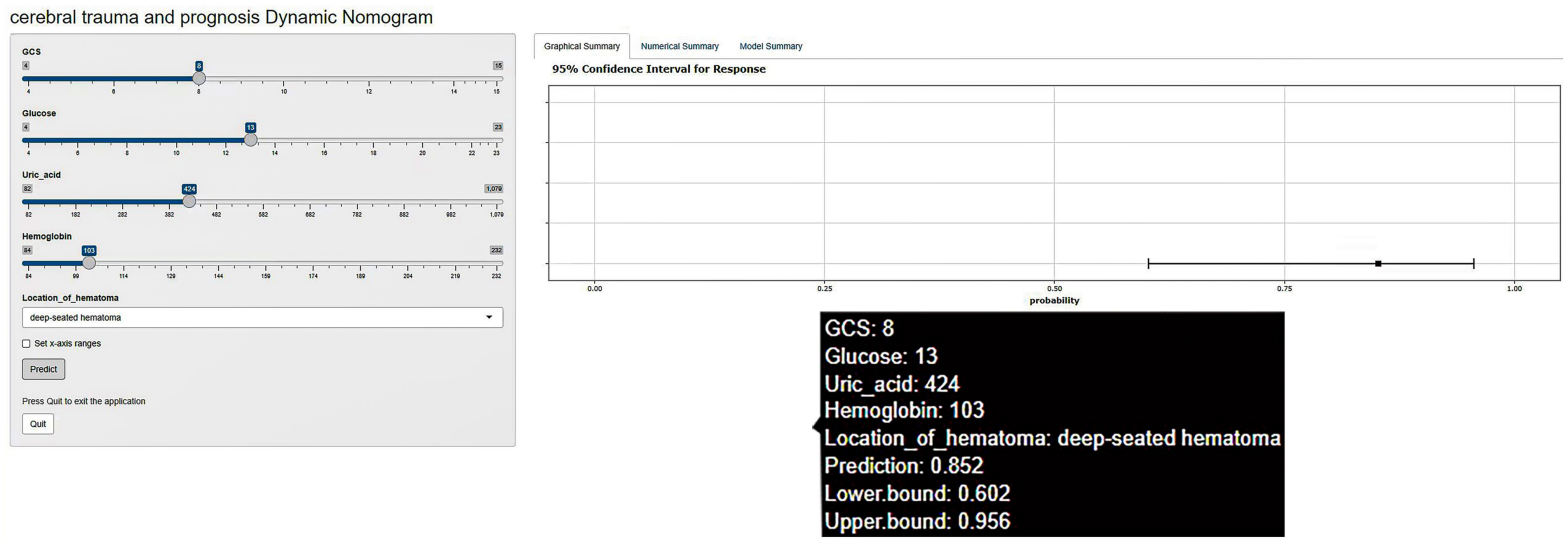
Dynamic nomogram of poor prognosis for intracerebral hemorrhage.

To visualize the prediction results, a nomogram was developed after performing a logistic regression analysis that predicts the individualized risk of an adverse outcome within 90 days after intracerebral hemorrhage. The user simply enters the patient information on the left side of the interface, and the risk of adverse prognosis within 90 days for that patient is displayed on the right side (Figure 3). For example, the first patient in the training set, a patient with a deep hematoma with a GCS score of 8, a glucose level of 13 mmol/L, a uric acid level of 424 μmol/L, and a hemoglobin of 103 g/L, had a predicted probability of adverse prognosis of 85.2% (95% CI: 60.2, 95.6%).

### Evaluation and internal testing of nomogram performance

**Figure 4:**
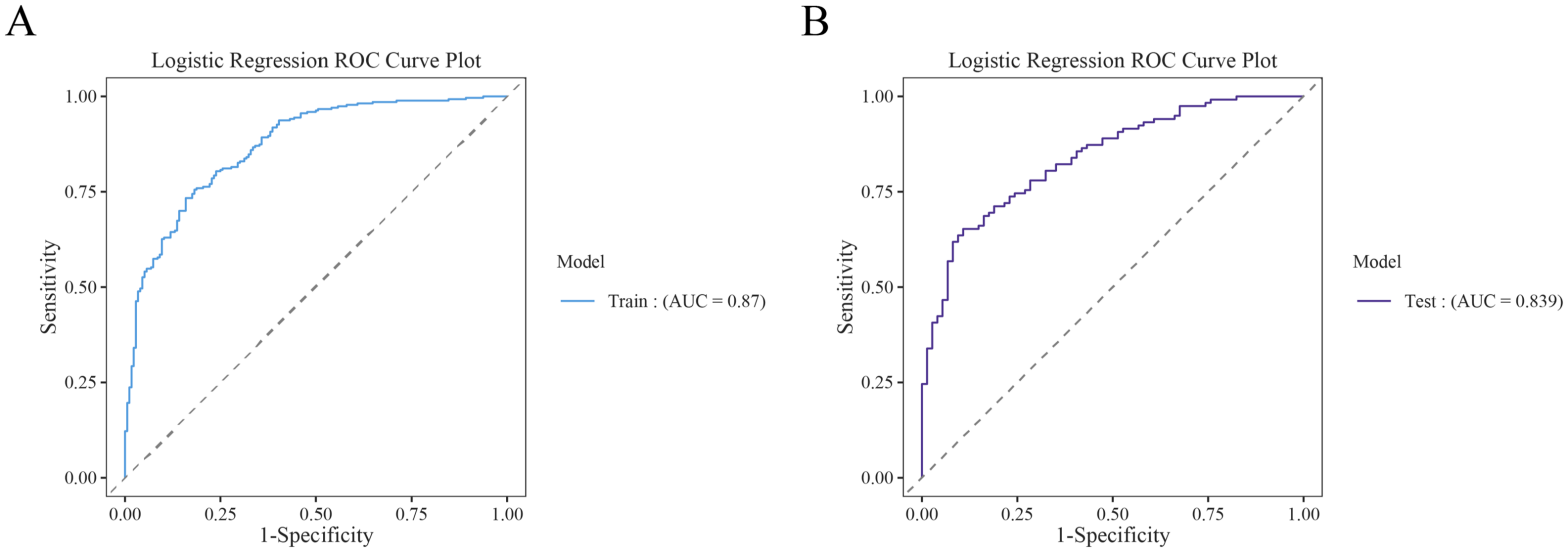
Receiver operating characteristic (ROC) curves for the training and test cohorts. **(A)** ROC curves for the training set. **(B)** ROC curves for the test set.

**Figure 5:**
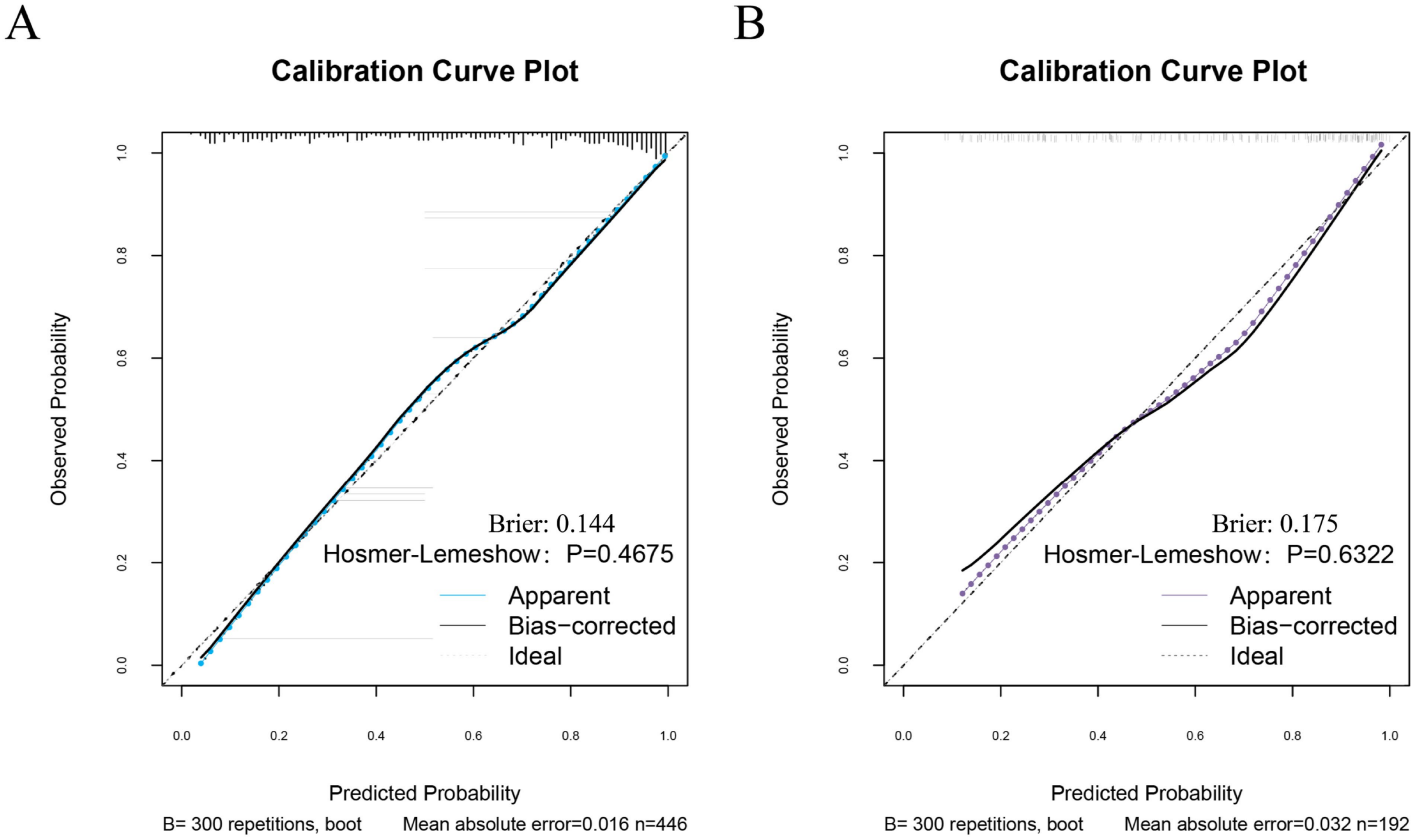
Calibration curves for the training and test sets. **(A)** Calibration curves for the training set. **(B)** Calibration curve of the test set.

**Figure 6:**
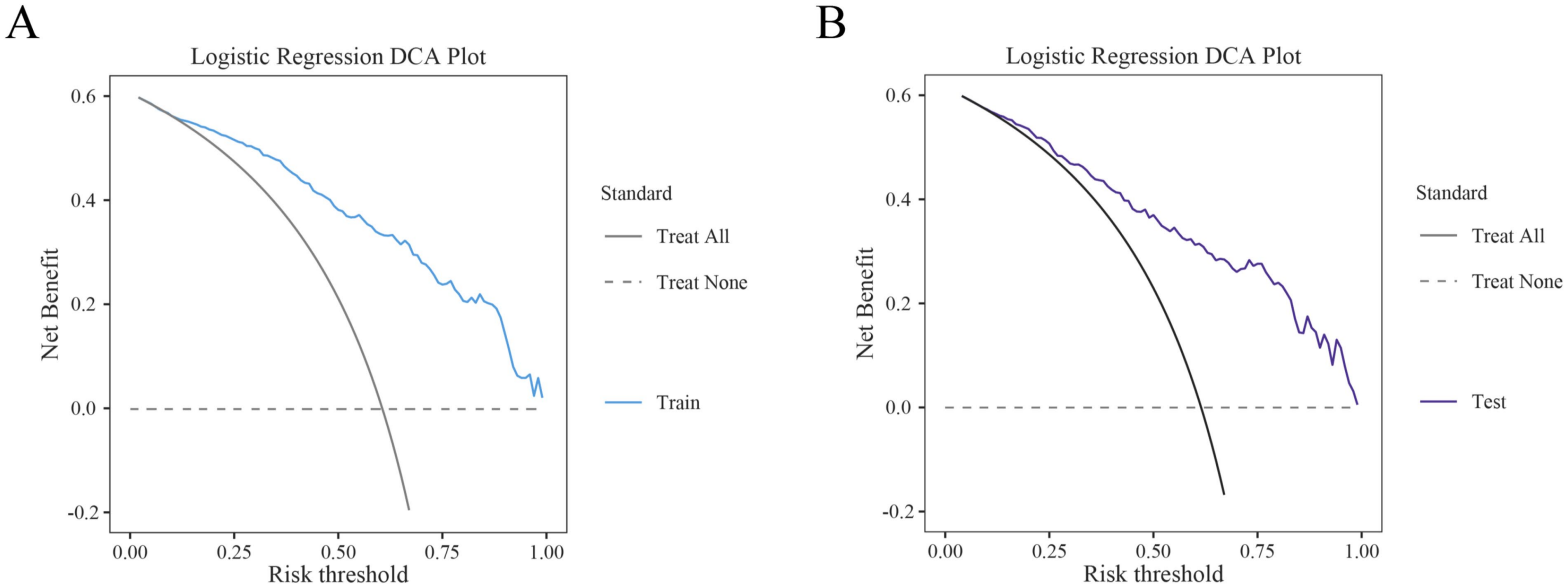
Decision curve analysis (DCA) of the training and test sets. **(A)** DCA of the training set. **(B)** DCA of the test set.

The performance of the nomogram for poor prognosis following intracerebral hemorrhage was evaluated using ROC curves, calibration curves, and clinical decision curves. In the ROC curve analysis (Figures 4A,B), the AUC for the training set and the validation set was 0.87 and 0.839, respectively, demonstrating the model’s good discriminatory ability. The calibration curve assessed the consistency between predicted probabilities and actual outcomes, with the gray solid line representing the ideal model’s perfect prediction and the dashed line showing the actual performance. The results (Figure 5) indicated that the dashed lines for both the training and validation sets almost perfectly matched the gray solid line. Hosmer-Lemeshow test results with *p*-values greater than 0.05 (training set *p* = 0.4675, validation set *p* = 0.6322) confirmed the model’s accurate calibration. In addition, the model’s Brier scores are all below 0.25, indicating that it performs well in the prediction process and has high prediction accuracy. In this study, the clinical validity of the model was assessed in the training and validation sets using DCA. The results show that the model has good clinical applicability in predicting the risk of poor prognosis in patients with cerebral hemorrhage. In particular, in the test set, the model was able to provide significant net clinical benefit over a wide and practical range of decision thresholds (Figure 6).

### External validation of the model

**Figure 7:**
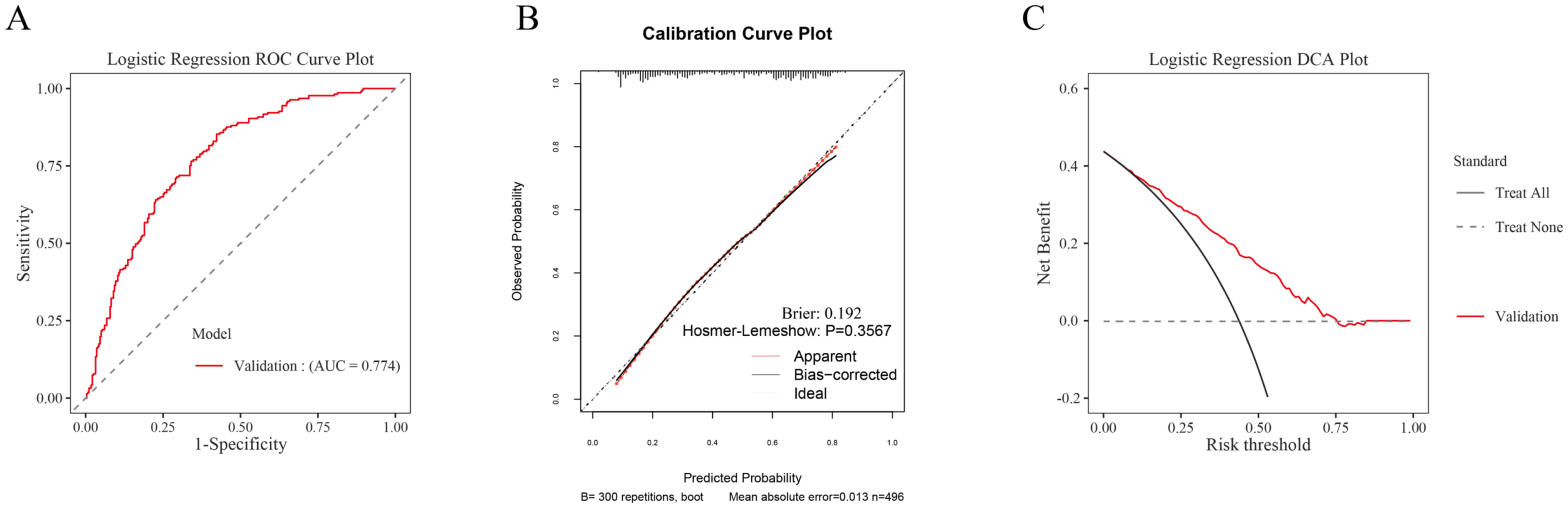
Aspects of model performance in external validation. **(A)** ROC for external validation sets. **(B)** Calibration for external validation sets. **(C)** DCA for external validation sets.

To assess the model’s generalizability, an external validation was conducted using the clinical data of 496 participants at another center (Figure 7). The results showed that the ROC curve area under the curve (AUC) for the post-hemorrhagic adverse outcome prediction model was 0.774, demonstrating good discriminative ability (Figure 7A). Additionally, the calibration curve for external validation showed high concordance between predicted and observed values, with a Hosmer-Lemeshow test *p*-value of 0.3567 (*p* > 0.05), indicating accurate model calibration (Figure 7B). The clinical decision curves (DCA) used for external validation indicated that the model could effectively predict the net benefit of adverse outcome risks across a decision threshold probability range of 10–75% (Figure 7C).

## Discussion

In this study, a user-friendly web-based nomogram was developed to predict the probability of poor outcomes within 90 days for patients with intracranial hemorrhage in neurosurgical wards. The baseline demographic, radiological, and laboratory findings at admission were collected. Then using LASSO and multivariate logistic regression analyses, GCS, glucose, uric acid, hemoglobin, and hematoma location were identified as independent risk factors and the nomogram was constructed^1^. The nomogram demonstrated excellent discrimination, calibration, and clinical utility, performing robustly in both the test set and external validation cohorts. Utilizing this nomogram, clinicians can quickly assess patients’ risks of adverse outcomes, thereby facilitating targeted management decisions. Thus, this tool helps those at high risk for poor prognosis to benefit from more intensive monitoring, preventive interventions, and early treatment.

In 2019, approximately 12.2 million new cases of stroke were recorded globally, along with 6.55 million stroke-related deaths, and about 101 million people suffered from post-stroke sequelae (26). Stroke is primarily categorized into ischemic and hemorrhagic types. Due to the relatively high prevalence of ischemic stroke, ischemic stroke is currently being studied in greater depth than hemorrhagic stroke, particularly regarding prognostic factors and patient management (25, 26). Numerous studies have identified several factors associated with poor prognosis. In a retrospective study involving 422 patients with ischemic stroke, researchers developed a predictive nomogram based on age, admission NIHSS score, history of COPD, and white blood cell count (WBC). This model demonstrated high predictive accuracy across training, testing, and validation sets (Training set AUC: 0.958 [95% CI: 0.918–0.997]; Testing set AUC: 0.962 [95% CI: 0.898, 1.000]) (16). Furthermore, Jin et al. (27) revealed in a large study that the LASSO regression-developed nomogram effectively predicts in-hospital adverse outcomes in ischemic stroke patients (C-index: 0.809), performing well in measures such as Integrated Discrimination Improvement (IDI), Net Reclassification Index (NRI), calibration curves, and DCA. Research on risk factors for patients with hemorrhagic stroke remains relatively scarce. However, given the severe clinical consequences that hemorrhagic strokes can entail, such research cannot be overlooked. The methodology that has been successful in ischemic stroke studies has now spurred interest in the scientific community to apply similar models to study hemorrhagic stroke. In a 2023 study involving 269 patients with intracerebral hemorrhage, researchers analyzed risk factors for prognosis 90 days post-hemorrhage. The results identified the GCS, the National Institutes of Health Stroke Scale (NIHSS), and the volume of the primary hematoma as independent risk factors (11). Although the predictive model achieved an area under the curve (AUC) of 87.8% (95% CI: 83.4, 92.2%), the study neither assessed the model’s calibration and clinical applicability nor developed a clinically applicable nomogram. Finally, the researchers acknowledged that due to the limited sample size, the study faced some limitations in the generalizability and reliability of its results.

This study identified five independent risk factors associated with poor prognosis in patients with intracerebral hemorrhage, including GCS, glucose, uric acid, hemoglobin, and hematoma location. Identifying these factors optimizes the assessment process and improves the utility and convenience of clinical applications, especially in creating web-based nomograms. Further, this study explored the possible mechanisms of association of these factors with poor prognosis. The present study reiterates that the GCS score is an independent risk factor for poor prognosis in patients with intracerebral hemorrhage. This is consistent with previous results (9). Patients with intracerebral hemorrhage with low GCS scores usually face more severe prognostic challenges due to increased intracranial pressure leading to neuronal injury, increased metabolic and inflammatory responses, and systemic complications. In the multifactorial logistic regression of this study, the OR for blood glucose was 1.12, indicating that the risk of poor prognosis in patients with primary cerebral hemorrhage increased as the blood glucose values increased. This hyperglycemia is part of the body’s response to acute stress and is prevalent in patients with intracerebral hemorrhage. Kongwad et al. (28) reported that hyperglycemia was directly associated with the prognosis of intracerebral hemorrhage. In hyperglycemia, plasma kallikrein (PK) exacerbates hematoma expansion after intracerebral hemorrhage by enhancing collagen binding and inhibiting collagen-induced platelet aggregation, further affecting patient prognosis (29). In addition, an animal experiment using a proteomic approach revealed the role of hyperglycemia in intracerebral hemorrhage and found a significant increase in neuronal apoptosis around hematomas in hyperglycemic rats. This may be related to the hyperglycemia-induced enhancement of oxygen radical synthesis and downregulation of superoxide dismutase activity, which promotes oxidative stress, a response that is extremely detrimental to neuronal survival (30). Similar to the oxidative stress induced by blood glucose, uric acid may also cause nerve damage through this mechanism. Uric acid has both antioxidant and pro-oxidant properties. At normal levels, uric acid acts as an antioxidant, trapping free radicals and protecting cells from oxidative damage. However, in hyperuricemia, uric acid may upset the redox balance of the body through multiple pathways (31). Excess free radicals from oxidative stress can attack unsaturated fatty acids in cell membranes and trigger the lipid peroxidation reaction chain to produce more free radicals and toxic peroxidation products such as malondialdehyde (MDA) (32). These products can further damage cell membranes and affect the integrity and function of neuronal cells (33). Also, the inflammatory storm caused by high uric acid levels affects intracerebral hemorrhage prognosis. Inflammation is a common pathological process after ICH that can lead to increased brain tissue damage. Uric acid can activate immune cells, such as monocytes and macrophages, to release inflammatory mediators and promote the inflammatory process, thus affecting the prognosis of ICH (8, 32). A German cohort study showed that anemia appears to be a significant predictor of poor functional outcome (OR: 3.0; *p* < 0.01) (34). This may be related to decreased cerebral oxygen delivery. Anemia decreases hemoglobin concentration, which reduces oxygen delivery to brain tissue. This hypoxic state increases brain cell damage, especially around the hemorrhagic area exacerbating hypoxic brain damage, which supports our study (35). The location of the hematoma is critical to the neurosurgeon’s treatment decisions. Deep hematomas located in the thalamus, basal ganglia, and brainstem can affect basic life functions such as respiration, heart rate regulation, and state of consciousness. Damage to these critical areas can severely impact life support systems, leading to serious clinical consequences (36). In addition, due to the unique location of deep hematomas surrounded by vital tissues, surgical intervention to remove the hematoma or decompress it is risky and technically demanding (37, 38). As a result, this complexity often limits the feasibility of rapid surgical intervention and complicates the management of such hematomas.

A new prognostic prediction model for ICH was developed to address the limitations of existing models in terms of complexity and usability. First, the model simplified data input requirements by using only information that has been commonly used and readily available in the clinic. Second, the model was validated internally and externally with a large number of data pairs to ensure its accuracy and broad applicability. Finally, to improve the ease of clinical operation, a user-friendly web platform that allowed medical staff to easily enter data and obtain immediate prognostic information was also developed. However, this study has some shortcomings. Although we tried our best to collect all the variables related to the prognosis of intracerebral hemorrhage, certain key variables, such as NIHSS scores, were not evaluated in routine diagnostic practices, which led to a certain selection bias. In addition, this study involved only two centers, and in the future, we hope to further improve the generalization ability of the model through future studies in more centers. Finally, this study only included patients with cerebral hemorrhage in neurosurgery and did not cover patients with cerebral hemorrhage in other departments, which may have a selection bias. In the future, we will expand the sample source to enhance the reliability of the study results.

## Conclusion

In summary, our study identified the independent risk factors for poor prognosis at 90 days in patients with intracerebral hemorrhage, including GCS, glucose, uric acid, hemoglobin, and hematoma location. Using LASSO and multivariate logistic regression algorithms, we successfully developed and validated a column chart that accurately predicts prognosis at 90 days in this patient population. Our nomogram showed good discrimination, calibration, and net clinical benefit and performed well in an external validation set, emphasizing its accuracy and utility in clinical practice. The model significantly improved patient recovery outcomes by accurately identifying individuals at high risk of poor prognosis after intracerebral hemorrhage. By prioritizing the placement of these individuals in the neurointensive care unit and implementing early individualized management strategies, including precise blood pressure control, aggressive complication management, and early physical rehabilitation, the patient’s outcomes were substantially improved. However, external validation and prospective studies at additional centers are needed to confirm the validity of this column chart.

## Data Availability

The data analyzed in this study will be made available upon reasonable request. Requests to access these datasets should be directed to HG, gaohongzhi@fjmu.edu.cn.

## Footnotes

^1^ https://stroke2024.shinyapps.io/DynNomapp/

## References

[1] Wang S Zou X-L Wu L-X Zhou H-F Xiao L Yao T. Epidemiology of intracerebral hemorrhage: a systematic review and meta-analysis. Front Neurol. (2022) 13:915813. doi: 10.3389/fneur.2022.915813, PMID: 36188383 PMC9523083

[2] Zhang L-F Yang J Hong Z Yuan G-G Zhou B-F Zhao L-C. Collaborative Group of China Multicenter Study of cardiovascular epidemiology. Proportion of different subtypes of stroke in China. Stroke. (2003) 34:2091–6. doi: 10.1161/01.STR.0000087149.42294.8C, PMID: 12907817

[3] Strilciuc S Grad DA Radu C Chira D Stan A Ungureanu M. The economic burden of stroke: a systematic review of cost of illness studies. J Med Life. (2021) 14:606–19. doi: 10.25122/jml-2021-0361, PMID: 35027963 PMC8742896

[4] Wang W Jiang B Sun H Ru X Sun D Wang L. Prevalence, incidence, and mortality of stroke in China: results from a Nationwide population-based survey of 480 687 adults. Circulation. (2017) 135:759–71. doi: 10.1161/CIRCULATIONAHA.116.025250, PMID: 28052979

[5] Huang C Liu X Zhao G Qian W Zhang Y Zhang W. Neuroendoscopic surgery for brainstem hemorrhage: technical notes and preliminary clinical results. Clin Neurol Neurosurg. (2024) 246:108576. doi: 10.1016/j.clineuro.2024.108576, PMID: 39366160

[6] Cui M Tang X Xiong W Deng Y Yang Q. Feasibility study of endoscopic surgery for spontaneous intracerebral hemorrhage with large hematoma: a comparison with craniotomy using propensity score matching analysis. Neurocrit Care. (2024). doi: 10.1007/s12028-024-02085-0, PMID: 39192100 PMC11950029

[7] GBD 2021. Stroke risk factor collaborators. Global, regional, and national burden of stroke and its risk factors, 1990-2021: A systematic analysis for the Global Burden of Disease Study. Lancet Neurol. (2024) 23. doi: 10.1016/S1474-4422(24)00369-7, PMID: 39304265

[8] Cheng X Hu D Wang C Lu T Ning Z Li K. Plasma inflammation markers linked to complications and outcomes after spontaneous intracerebral hemorrhage. J Proteome Res. (2024) 23:4369–83. doi: 10.1021/acs.jproteome.4c00311, PMID: 39225497

[9] Wu X Liu J Tian D Chen J Li H. Associations of serum Dickkopf-1 levels with disease severity and 90-day prognosis after spontaneous intracerebral hemorrhage: results from the prospective cohort study. Neurosurg Rev. (2024) 47:528. doi: 10.1007/s10143-024-02755-9, PMID: 39227406

[10] Misra S Kawamura Y Singh P Sengupta S Nath M Rahman Z. Prognostic biomarkers of intracerebral hemorrhage identified using targeted proteomics and machine learning algorithms. PLoS One. (2024) 19:e0296616. doi: 10.1371/journal.pone.0296616, PMID: 38829877 PMC11146689

[11] Feng H Wang X Wang W Zhao X. Risk factors and a prediction model for the prognosis of intracerebral hemorrhage using cerebral microhemorrhage and clinical factors. Front Neurol. (2023) 14:1268627. doi: 10.3389/fneur.2023.1268627, PMID: 38073656 PMC10701734

[12] Song X Zhang H Han Y Lou S Zhao E Dong Y. Based on hematoma and perihematomal tissue NCCT imaging radiomics predicts early clinical outcome of conservatively treated spontaneous cerebral hemorrhage. Sci Rep. (2024) 14:18546. doi: 10.1038/s41598-024-69249-y, PMID: 39122887 PMC11315882

[13] Zhao C Lu Z Hua B Yue J Yang Q Ni H. Predictive value of current perception threshold for prognosis of pulsed radiofrequency in patients with acute herpetic neuralgia. J Pain Res. (2024) 17:3241–53. doi: 10.2147/JPR.S472535, PMID: 39371492 PMC11456298

[14] Li S Feng Q Wang J Wu B Qiu W Zhuang Y. A machine learning model based on CT imaging metrics and clinical features to predict the risk of hospital-acquired pneumonia after traumatic brain injury. Infect Drug Resist. (2024) 17:3863–77. doi: 10.2147/IDR.S473825, PMID: 39253609 PMC11382661

[15] Ji Z Ye W Wen X Zhao X Li N. Predicting intracerebral hemorrhage expansion with inflammation indices, non-contrast computed tomography signs and computed tomography angiography spot sign. Neuropsychiatr Dis Treat. (2024) 20:1879–87. doi: 10.2147/NDT.S475550, PMID: 39376667 PMC11457768

[16] Zhou L Wu Y Wang J Wu H Tan Y Chen X. Development of a predictive nomogram for intra-hospital mortality in acute ischemic stroke patients using LASSO regression. Clin Interv Aging. (2024) 19:1423–36. doi: 10.2147/CIA.S471885, PMID: 39139210 PMC11321337

[17] Sawada J Katayama T Kikuchi-Takeguchi S Kano K Saito M Mitsui N. Clinical features and prognostic factors of patients with cancer-associated stroke. Neurol Sci. (2024) 45:2747–57. doi: 10.1007/s10072-024-07332-y 38267601

[18] Biffi A Anderson CD Jagiella JM Schmidt H Kissela B Hansen BM. APOE genotype predicts extent of bleeding and outcome in lobar intracerebral hemorrhage. Lancet Neurol. (2011) 10:702–9. doi: 10.1016/S1474-4422(11)70148-X, PMID: 21741316 PMC3153411

[19] Xu X Chen X Zhang J Zheng Y Sun G Yu X. Comparison of the Tada formula with software slicer: precise and low-cost method for volume assessment of intracerebral hematoma. Stroke. (2014) 45:3433–5. doi: 10.1161/STROKEAHA.114.007095, PMID: 25316277

[20] Zhao L Cui M Yang S Zhou H Li M. Systemic inflammatory indicators and risk of incident metabolically unhealthy phenotype. J Inflamm Res. (2024) 17:6905–16. doi: 10.2147/JIR.S474201, PMID: 39372594 PMC11451455

[21] Rodriguez-Luna D Coscojuela P Rubiera M Hill MD Dowlatshahi D Aviv RI. Ultraearly hematoma growth in active intracerebral hemorrhage. Neurology. (2016) 87:357–64. doi: 10.1212/WNL.0000000000002897, PMID: 27343067 PMC4977111

[22] Wang W-J Lu J-J Liu L-P Jia J-K Zhao X-Q. Ultraearly hematoma growth in acute spontaneous intracerebral hemorrhage predicts early and long-term poor clinical outcomes: a prospective. Observational Cohort Study Front Neurol. (2021) 12:747551. doi: 10.3389/fneur.2021.747551, PMID: 34975715 PMC8714734

[23] Li S Wang Y Wang W Zhang Q Wang A Zhao X. Stress hyperglycemia is predictive of clinical outcomes in patients with spontaneous intracerebral hemorrhage. BMC Neurol. (2022) 22:236. doi: 10.1186/s12883-022-02760-9, PMID: 35761206 PMC9235136

[24] Zhang W Huang Z Huang Y Dai Y Lu H Chen Z. Factors influencing recurrence after an ischemic stroke vary by sex. Neurol Res. (2023) 45:827–34. doi: 10.1080/01616412.2023.2211433, PMID: 37170802

[25] Huang Z Yang D Huang Y Xu G Liu X. Letter to the editor: selection of appropriate statistical methods for prediction model. Hepatology. (2022) 75:1348–9. doi: 10.1002/hep.32371, PMID: 35092078

[26] GBD 2019 Stroke Collaborators. Global, regional, and national burden of stroke and its risk factors, 1990-2019: a systematic analysis for the global burden of disease study 2019. Lancet Neurol. (2021) 20:795–820. doi: 10.1016/S1474-4422(21)00252-0 34487721 PMC8443449

[27] Jin G Hu W Zeng L Diao M Chen H Chen J. Development and verification of a nomogram for predicting short-term mortality in elderly ischemic stroke populations. Sci Rep. (2023) 13:12580. doi: 10.1038/s41598-023-39781-4, PMID: 37537270 PMC10400586

[28] Kongwad LI Hegde A Menon G Nair R. Influence of admission blood glucose in predicting outcome in patients with spontaneous intracerebral hematoma. Front Neurol. (2018) 9:725. doi: 10.3389/fneur.2018.00725, PMID: 30210444 PMC6121104

[29] Liu J Gao B-B Clermont AC Blair P Chilcote TJ Sinha S. Hyperglycemia induced cerebral hematoma expansion is mediated by plasma Kallikrein. Nat Med. (2011) 17:206–10. doi: 10.1038/nm.2295, PMID: 21258336 PMC3038677

[30] Chiu C-D Chen T-Y Chin L-T Shen C-C Huo J Ma S-Y. Investigation of the effect of hyperglycemia on intracerebral hemorrhage by proteomic approaches. Proteomics. (2012) 12:113–23. doi: 10.1002/pmic.201100256, PMID: 22065606

[31] Kang D-H Ha S-K. Uric acid puzzle: dual role as anti-oxidantand pro-oxidant. Electrolyte Blood Press. (2014) 12:1–6. doi: 10.5049/EBP.2014.12.1.1, PMID: 25061467 PMC4105384

[32] Yan K-X Ge B-J Sang R Zhao P Liu X-M Yu M-H. Taraxasterol attenuates zearalenone-induced kidney damage in mice by modulating oxidative stress and endoplasmic reticulum stress. Ecotoxicol Environ Saf. (2024) 285:117093. doi: 10.1016/j.ecoenv.2024.117093, PMID: 39317070

[33] Pannangrong W Nillert N Boonyarat C Welbat JU Yannasithinon S Choowong-In P. Clausena harmandiana root extract ameliorates Aβ1-42 induced cognitive deficits, oxidative stress, and apoptosis in rats. BMC Complement Med Ther. (2024) 24:364. doi: 10.1186/s12906-024-04662-4, PMID: 39390478 PMC11465876

[34] Kuramatsu JB Gerner ST Lücking H Kloska SP Schellinger PD Köhrmann M. Anemia is an independent prognostic factor in intracerebral hemorrhage: an observational cohort study. Crit Care. (2013) 17:R148. doi: 10.1186/cc12827, PMID: 23880122 PMC4057052

[35] Poyraz FC Boehme A Cottarelli A Eisler L MSV E Ghoshal S. Red blood cell transfusions are not associated with incident complications or poor outcomes in patients with intracerebral hemorrhage. J Am Heart Assoc. (2023) 12:e028816. doi: 10.1161/JAHA.122.028816, PMID: 37232240 PMC10381991

[36] Sattari SA Yang W Feghali J Hung A Xu R Tamargo RJ. Management and outcome predictors of patients with ruptured deep-seated brain arteriovenous malformations. J Neurosurg. (2023) 140:755–63. doi: 10.3171/2023.6.JNS23459, PMID: 37721414

[37] Watanabe G Conching A Ogasawara C Chavda V Bin-Alamer O Haider AS. Bilateral basal ganglia hemorrhage: a systematic review of etiologies, management strategies, and clinical outcomes. Neurosurg Rev. (2023) 46:135. doi: 10.1007/s10143-023-02044-x, PMID: 37273079 PMC10240133

[38] Wang A Sun Z Zhang W He H Wang F. Efficacy and safety of endoscopic surgery versus craniotomy for hypertensive putamen hemorrhage. J Craniofac Surg. (2024) 35:1181–5. doi: 10.1097/SCS.0000000000010105, PMID: 38595184

