# Supplementary material for: Development and external validation of a dynamic nomogram for predicting the risk of functional outcome after 90 days in patients with acute intracerebral hemorrhage
Source: Front Neurol. 2025 Jan 29;16:1519091. doi: 10.3389/fneur.2025.1519091 (PMC11816111; doi:10.3389/fneur.2025.1519091)
Supplement: Supplementary file 1 [file Table_1.DOC]

Supplementary Table 1 Baseline characteristics of poor prognosis for intracerebral hemorrhage stratified by sex

| Variable Names | Overall | Male | Female | *P* |
| --- | --- | --- | --- | --- |
|  | N=388 | N=263 | N=125 |  |
| Age (years) | 58.46±10.64 | 58±11.33 | 59.42±8.98 | 0.22 |
| GCS | 8.91±2.63 | 8.74±2.65 | 9.27±2.56 | 0.06 |
| Temperature（℃） | 36.95±0.68 | 36.93±0.65 | 36.98±0.74 | 0.47 |
| Systolic blood pressure (mmHg) | 165.54±20.58 | 166.02±21.03 | 164.52±19.63 | 0.5 |
| Diastolic blood pressure (mmHg) | 91.93±15.36 | 93.25±15.66 | 89.13±14.37 | 0.01 |
| Glucose (mmol/L) | 8.68±3.43 | 8.6±3.36 | 8.86±3.57 | 0.47 |
| Uric acid (umol/L) | 313.26±135.5 | 343.01±141.69 | 250.68±95.17 | <0.01 |
| Albumin (g/L) | 41.1±4.28 | 41.01±4.29 | 41.3±4.27 | 0.54 |
| Leucocyte | 10.45±4.27 | 10.46±4.34 | 10.42±4.15 | 0.92 |
| Hemoglobin (g/L) | 152.4±23.97 | 158.51±23.06 | 139.54±20.59 | <0.01 |
| NLR | 10.75±8.65 | 10.21±8.64 | 11.88±8.57 | 0.07 |
| LMR | 3.27±3.14 | 2.98±2.05 | 3.87±4.61 | 0.01 |
| PLR | 189.71±117.58 | 174.04±107.66 | 222.67±130.56 | <0.01 |
| PT | 11.83±3.17 | 11.95±3.35 | 11.56±2.74 | 0.26 |
| INR | 1.05±0.29 | 1.06±0.31 | 1.02±0.24 | 0.24 |
| APTT | 25.52±5.63 | 25.94±5.61 | 24.63±5.59 | 0.03 |
| D-dimer | 2.95±0.93 | 2.95±0.99 | 2.95±0.78 | 0.99 |
| TT | 16.85±2.64 | 16.79±2.68 | 16.98±2.58 | 0.51 |
| FIB | 1.76±7.2 | 1.96±8.59 | 1.35±2.38 | 0.44 |
| Bleeding volume (mL) | 33.35±25.69 | 35.04±26.91 | 29.78±22.62 | 0.06 |
| UHG | 10.64±8.91 | 11.14±9.13 | 9.57±8.36 | 0.11 |
| Hypertension (%) |  |  |  | 0.72 |
| No | 118 (30.41) | 82 (31.18) | 36 (28.80) |  |
| Yes | 270 (69.59) | 181 (68.82) | 89 (71.20) |  |
| Diabetes (%) |  |  |  | 0.78 |
| No | 312 (80.41) | 213 (80.99) | 99 (79.20) |  |
| Yes | 76 (19.59) | 50 (19.01) | 26 (20.80) |  |
| Smoking (%) |  |  |  | <0.01 |
| No | 339 (87.37) | 214 (81.37) | 125 (100.00) |  |
| Yes | 49 (12.63) | 49 (18.63) | 0 (0.00) |  |
| Drinking (%) |  |  |  | 0.03 |
| No | 349 (89.95) | 230 (87.45) | 119 (95.20) |  |
| Yes | 39 (10.05) | 33 (12.55) | 6 (4.80) |  |
| History of anticoagulant use (%) |  |  |  | 0.73 |
| No | 364 (93.81) | 248 (94.30) | 116 (92.80) |  |
| Yes | 24 (6.19) | 15 (5.70) | 9 (7.20) |  |
| Tracheotomy (%) |  |  |  | 0.24 |
| No | 288 (74.23) | 190 (72.24) | 98 (78.40) |  |
| Yes | 100 (25.77) | 73 (27.76) | 27 (21.60) |  |
| Lateral ventricular hemorrhage (%) |  |  |  | 0.72 |
| No | 270 (69.59) | 181 (68.82) | 89 (71.20) |  |
| Yes | 118 (30.41) | 82 (31.18) | 36 (28.80) |  |
| Location of hematoma (%) |  |  |  | 0.36 |
| deep-seated hematoma | 54 (13.92) | 40 (15.21) | 14 (11.20) |  |
| superficial hematoma | 334 (86.08) | 223 (84.79) | 111 (88.80) |  |
| Centerline shift(%) |  |  |  | 0.9 |
| No | 264 (68.04) | 180 (68.44) | 84 (67.20) |  |
| Yes | 124 (31.96) | 83 (31.56) | 41 (32.80) |  |
| Surgeries(%) |  |  |  | 1.00 |
| No | 136 (35.05) | 92 (34.98) | 44 (35.20) |  |
| Yes | 252 (64.95) | 171 (65.02) | 81 (64.80) |  |
