# Supplementary material for: Development and external validation of a dynamic nomogram for predicting the risk of functional outcome after 90 days in patients with acute intracerebral hemorrhage
Source: Front Neurol. 2025 Jan 29;16:1519091. doi: 10.3389/fneur.2025.1519091 (PMC11816111; doi:10.3389/fneur.2025.1519091)
Supplement: Supplementary file 2 [file Table_2.DOC]

Supplementary Table 2 Table of coefficients for LASSO regression

| Coef Name | coeff_lamda |
| --- | --- |
| (Intercept) | -5.78259578757694 |
| Sex | 0 |
| Age | 8.19472868930285e-05 |
| Hypertension | -0.0517166646320285 |
| Diabetes | 0 |
| Smoking | 0.264160295583072 |
| Drinking | 0.23648978661272 |
| History of anticoagulant use | 0 |
| GCS | -0.354023690320462 |
| Temperature | 0.24777860859887 |
| Systolic blood pressure | 0.00920038237316219 |
| Diastolic blood pressure | 0 |
| Glucose | 0.0719634704875331 |
| Uric acid | 0.00134144835398749 |
| Albumin | -0.0365749784398408 |
| Leucocyte | 0.0403878947338687 |
| Hemoglobin | -0.0108241817711557 |
| NLR | 0 |
| LMR | 0.0205857633236788 |
| PLR | -0.000648652770123358 |
| PT | 0 |
| INR | 0 |
| APTT | 0.0178558702047577 |
| FIB | 0 |
| TT | -0.0420086049263084 |
| D-dimer | 0.000114763217685795 |
| Tracheotomy | 0.388969645402743 |
| Lateral ventricular hemorrhage | 0 |
| Location of hematoma | 0.896702442183526 |
| Centerline shift | 0.261358182241828 |
| Bleeding volume | 0.000129334164177837 |
| UHG | 0.0909809340125795 |
| Surgeries | 0.338006686267812 |
