# Supplementary material for: Development and external validation of a dynamic nomogram for predicting the risk of functional outcome after 90 days in patients with acute intracerebral hemorrhage
Source: Front Neurol. 2025 Jan 29;16:1519091. doi: 10.3389/fneur.2025.1519091 (PMC11816111; doi:10.3389/fneur.2025.1519091)
Supplement: Supplementary file 3 [file Table_3.DOC]

Supplementary Table 3 Variance inflation factors for different variables

| Variable Name | VIF |
| --- | --- |
| Location of hematoma | 1.04317328649949 |
| GCS | 1.02560990512104 |
| Glucose | 1.03098042154681 |
| Uric acid | 1.17536635091948 |
| Hemoglobin | 1.17083910015988 |
